# Supplementary material for: Chlamydomonas reinhardtii Alternates Peroxisomal Contents in Response to Trophic Conditions
Source: Cells. 2022 Sep 1;11(17):2724. doi: 10.3390/cells11172724 (PMC9454557; doi:10.3390/cells11172724)
Supplement: Supplementary file 1 [file cells-11-02724-s001.zip › cells-1882335-SUPPLEMENTARY.pdf]

Supplemental Materials

**Minimal Medium**	✓	✓	✓	✓	✓
**0.02 % (v/v) Tween 80**		✓	✓	✓	✓
**16 mM Acetic acid**			✓		
**5 mM palmitic acid (C16:0)**				✓	
**5 mM oleic acid (C18:1)**					✓

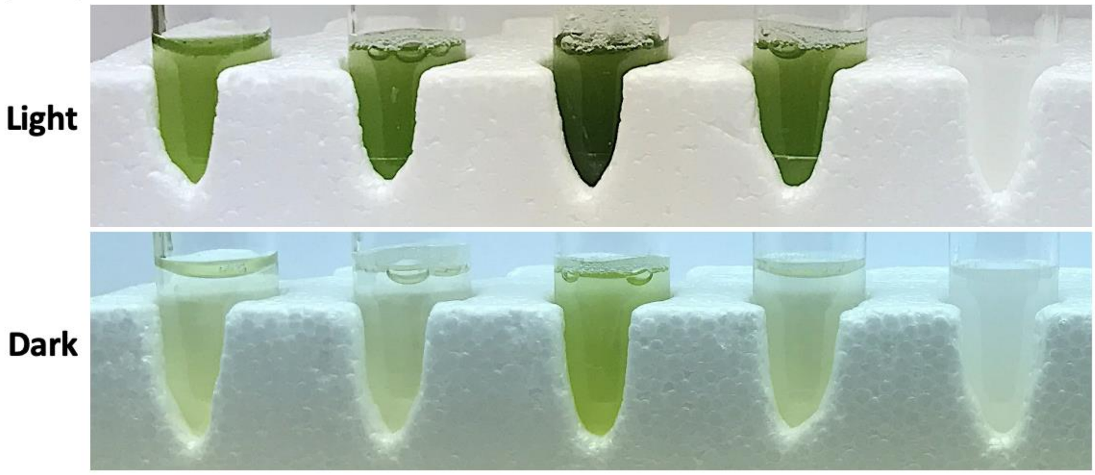

Figure S1 Oleic acid is toxic to *C. reinhardtii*. Direct comparison of growth with palmitic acid (C16:0) and oleic acid (C18:1). A *Chlamydomonas* strain CC5082 was cultured for 14 days in liquid MM (Minimal Medium) that contains Tween 80 and a different type of fatty acid, namely acetic acid, palmitic acid, and oleic acid. The cells were cultured with or without light. Notice that the cells proliferate in the dark only when acetic acid is in the medium. Cells are bleached out (died) with and without light when cultured in oleic acid.

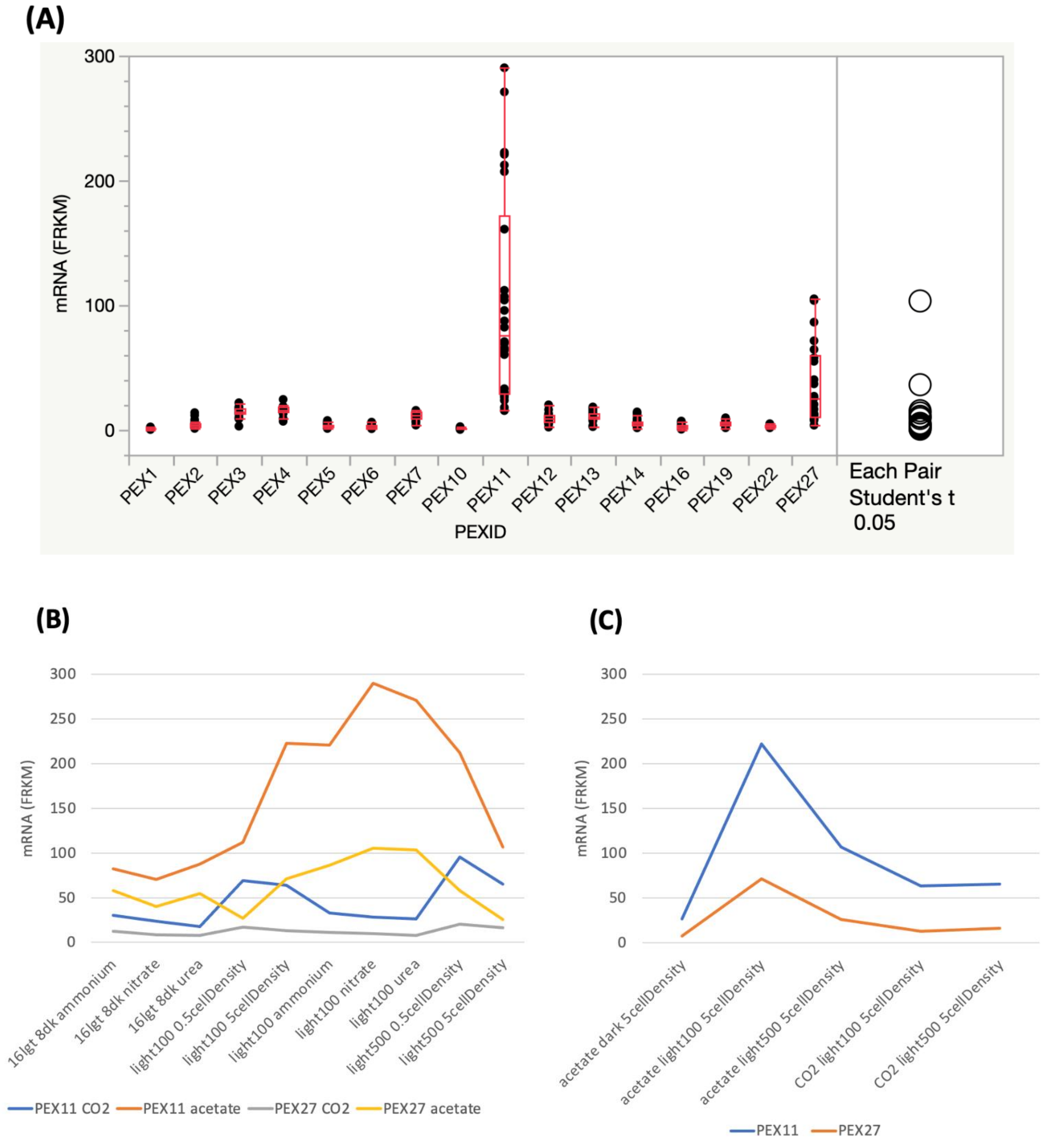

Figure S2 Expression of *Chlamydomonas* PEX genes, except PEX11 and PEX27, are expressed constantly at low levels in 26 different culture conditions: PEX11 and PEX27 are highly expressed in all culture conditions and upregulated by acetate. RNA-Seq data in Phytozome (https://phytozome-next.jgi.doe.gov) were analyzed. The data covers all 16 PEX genes and 26 different culture conditions, and the gene expressions were quantified as FRKM (fragments per kilobase of exon per million mapped reads) in the data. **(A)** Box plots of each gene (red). The gene expression levels in each culture condition were plotted (black dots) together with the result of each pair Student's t-test with P = 0.05 (circles). The analysis found that all PEX genes, except PEX11 and PEX27, are constantly expressed at similar (P > 0.05) and lower (1.1 < mean FRKM < 16.0) levels in all culture conditions. The expression of the PEx11 (mean FRKM 103.9) and PEX27 (mean FRKM 36.8) are significantly higher than the rest of the PEX genes (P < 0.001). **(B)** Comparison of gene expression levels of PEX11 and PEX27 in the cultures with and without acetate. The data of the

gene expressions of the PEX11 and PEX27 in 10 different culture conditions were plotted. Blue line: PEX11 expression with no acetate (CO_2 as a carbon source). Orange line: PEX11 expression with acetate. Gray line: PEX27 expression with no acetate (CO_2 as a carbon source). Yellow line: PEX27 expression with acetate. Notice that independent of the light conditions and nitrogen sources, the expression of the PEX11 and PEX27 are constantly higher when acetate is available than when only CO_2 is available as a carbon source. PEX11 is responsible for the division and proliferation of peroxisomes, and PEX27 is a peroxisomal membrane protein in yeast. This result supports the idea that the biogenesis of the peroxisomes is upregulated by acetate. **(C)** Comparison of gene expression levels of PEX11 and PEX27 in the cultures with different light conditions. The gene expression data of PEX11 and PEX27 in 5 different culture conditions were plotted. Blue line: PEX11 expression. Orange line: PEX27 expression. Notice that no clear conclusion can be made for the expression levels of the PEX11 and PEX27 on the light dependency. This suggests that the biogenesis of the peroxisomes may not rely on light conditions. The FRKMs of all 16 PEX genes in all 26 different culture conditions are available in Supplemental Table S1.

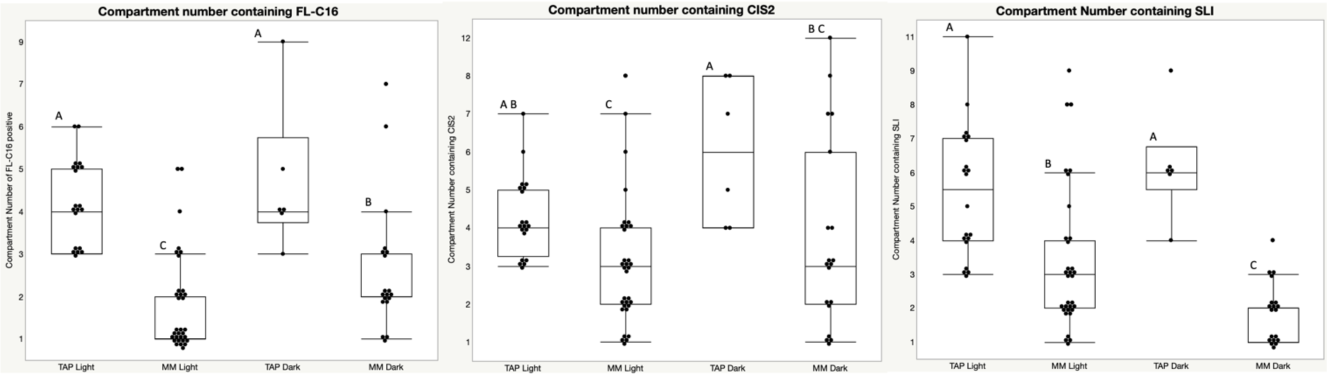

Figure S3 The number of compartments containing FL-C16, CIS2-RFP, or CFP-SLI is equally reduced when the cells are cultured without acetic acid. The number of compartments in an optical section detected by ComDet was presented as a box plot with individual data points. Notice that the number of compartments that contain FL-C16, CIS2-RFP, or CFP-SLI, respectively, reduces when the cells are cultured without acetic acid (MM) regardless of light conditions. . Letters on the top of each bar indicate mean comparisons for each pair using Student's t-test. Levels not connected by the same letter are significantly different ($p < 0.05$).
